# Selected Alternative Feed Additives Used to Manipulate the Rumen Microbiome

**DOI:** 10.3390/ani11061542

**Published:** 2021-05-25

**Authors:** Marta Michalak, Konrad Wojnarowski, Paulina Cholewińska, Natalia Szeligowska, Marcel Bawej, Jakub Pacoń

**Affiliations:** 1 Department of Animal Nutrition and Feed Management, Wroclaw University of Environmental and Life Sciences, 51-630 Wroclaw, Poland; 2 Institute of Animal Breeding, Wroclaw University of Environmental and Life Sciences, 51-630 Wroclaw, Poland; paulina.cholewinska@upwr.edu.pl (P.C.); 119635@student.upwr.edu.pl (N.S.); 112553@student.upwr.edu.pl (M.B.); 3 Department of Genetics, Wroclaw University of Environmental and Life Sciences, 51-630 Wroclaw, Poland; jakub.pacon@upwr.edu.pl

**Keywords:** feed additives, ruminants, plants, fermentation, probiotics, prebiotics

## Abstract

**Simple Summary:**

The continuous intensification of ruminant production drives towards the expansion of feed components and additives that are utilizes for the coverage of animal’s demand for nutrients. Additionally, in recent years, studies are focused on the investigation of how feed additives affect the microbiome of the digestive system in order to obtain improved performance and/or reduce methane emissions by ruminants. The use of additives such as algae, probiotics, fermented feed or essential oils can serve as an alternative to antibiotics or other synthetic compounds that may pose a danger to the environment.

**Abstract:**

In recent years, a boost in the ruminant population has been observed, and consequently, an increase in the animals’ demand for nutrients and methane emissions. Methane emission is generated during the microbial fermentation of feed in the rumen, and a percentage even up to 12% of the energy obtained by this process can be wasted. In addition, the use of antibiotics in animal husbandry is being increasingly restricted. restricted. As a result, there is a continuous search for innovative feed additives that can serve as alternatives to antibiotics, and will also be safe for both people and the environment. In the present review article, additives were selected on basis that, according to studies conducted so far, may positively affect the microbiome of the digestive system by improving indicators and/or reducing methane production. Among them, probiotics, prebiotics or their combination—synbiotics are at the forefront of research. However, additives in the form of algae or plant origin are also gaining ground in popularity, such as essential oils, fermented wheat straw or *Gelidium amansii*, due to their general recognition as safe (GRAS) for both humans and environment.

## 1. Introduction

The ruminant production sector plays an important role in world food chain and is constantly evolving. In the years 2015–2019 there was a rise in the world’s cattle population from 1.45 billion to over 1.5 billion, while in the same period, sheep and goats increased from 2.18 billion to 2.33 billion [1]. Intensification of ruminant production imposes the of animal production forces the constant expansion of the base of feed components and additives in order to cover the increasing demand of animals for nutrients, in particular, maintaining the balance between production and exploitation of the environment. Nowadays, the improvement of diet composition is a key factor used to improve the health status and welfare of animals [2] as well as to enhance productivity in livestock [3,4,5] and physical performance in athletic species [6,7,8,9].

One of the main problems in large-scale production of ruminants is methane emission, closely related to the gastrointestinal microbiome. Methane emission is induced during microbial fermentation of feed in the rumen, and up to 12% of the produced energy, potentially beneficial for host, can be lost, due to the use of organic acids necessary for the production of VFA by bacteria, i.e., formic acid, acetic acid [10,11,12,13].

Currently, there is a great interest in reducing the level of methane emissions in animal production through the use of feed additives that affect the composition of the microbiome of the digestive system of ruminants [14,15,16,17]. Feed additives are a class of nutrients that when added to the feed can influence, in a desired way, certain production parameters of animal. Some of these compounds have been widely used in animal nutrition for quite a long time, such as fodder yeast or linseed oil [18,19].

In recent years, many innovative ways to modify the microbiome of the ruminants’ digestive tract have emerged. Through the utilization of feed additives, such as fermented feed, probiotics, prebiotics, synbiotic or various types of residues from plant production [20,21,22] the level of production can be increased, health status of animals and quality of the derived products can be improved, as well as their negative impact on the environment can be minimized. In addition, nowadays, the development of new technologies such as metagenomics, metatranscriptomics or metabolomics along with the development of methods based on sequencing has allowed us to obtain previously unavailable data. This improves our ability to interpret and predict functional interactions, as well as antibiotic resistance or the dynamics of the development of the microbiome population with the possibility of using acquired knowledge in agriculture or environmental protection. Data obtained thanks to the application of the aforementioned modern methods create a new “space” for scientists dealing with this subject, which seems to be a natural environment for the further development of research aimed at a better understanding of the components and mechanisms related to the ruminant microbiome at various levels [13,14,20,21,22,23].

## 2. Fermented Feeds

The production of fermented feeds is related to the need to change and improve nutrient digestibility in of both ruminants and other animals. The use of the fermentation process on various types of feed, such as wheat straw, soybean meal or rapeseed, can increase the supplementation level of alternative feeds derived from agricultural waste. This will allow us to reduce the demand for conventional feedstuffs, and as a result, it opens the possibility to reduce land used in its production, which is difficult to obtain otherwise due to the changes in land usage [24,25].

**Figure 1:**
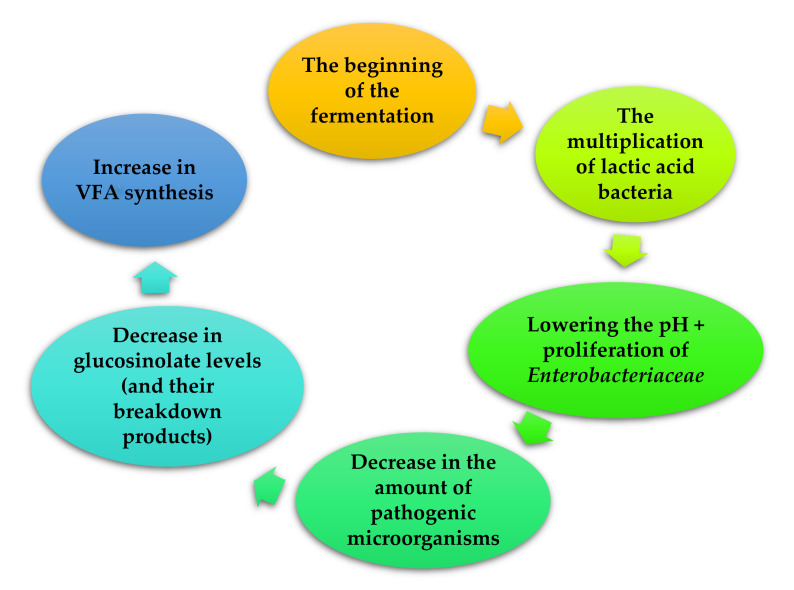
Simplified scheme of fermentation (based on [24,25,26]).

The aim of the fermentation process is to reduce the amount of anti-nutrients and toxins in the feed, degrade the crude fiber and reduce the level of lignin to increase the digestibility of the feed intake. It also aims to extend the period of use of the feed, and also changes the microbiological and nutritional properties of the mixture [26,27,28]. Additionally, fermentation as a biological method, is preferred over other methods because it produces minimal, harmful by-products and has low energy requirements [29,30]. There are many available feed fermentation techniques, such as: liquid fermentation, solid fermentation and ensilage. In the fermentation process, feed additives are decomposed by bacteria and/or yeast (Figure 1). The most commonly used yeast strains are: *Saccharomyces cerevisiae, Rhizopus oligosporus* and bacteria from the groups: *Lactobacillus, Bacillus Enterococcus* [28,31]. However, in the case of other fungi utilized in the process, these are mainly *Aspergillus* (e.g., *Aspergillus oryzae, Aspergillus niger*). The application of these microorganisms is related to the ability to produce enzymes such as: hemicellulase, pectanase, protease, amylase, lipase and phytase, as well as substances with bacteriostatic activity, such as lactic acid [28,32,33,34]. The use of the fermentation process significantly influences the level of pathogenic microorganisms in the feed. The forages fermented with *Pediococcus pentosaceus* at 20 °C and inoculated with *Salmonella typhimurium DT 104:30* were characterized by maintaining *S. typhimurium DT 104:30* for 72 h. However, no *S. typhimurium DT 104:30* was detected after incubation at 30 °C for 48 h. The decrease in the level of this pathogen was caused not only by an increase in the level of lactic acid but also by substances produced by *Pediococcus pentosaceus* possessing antimicrobial activity [28].

Fermented feeds are mainly used in juvenile ruminants, however, some types of them can also positively manipulate the microbiota of adult animals [28,35,36]. One example is the data presented by Azlan et al. [37] where it was shown that rice straw treated with *Aspergillus terreus* reduces the production of methane in ruminants by up to 32%. The obtained effect was related to the substance produced by *A. terreus*—levastatin, which inhibits the growth of *Methanobrevibacter smithii*, which resulted in reduced methane emissions. However, there was an increase in the level of *Ruminococcus albus*. The inhibition of the growth of methanogens is related to the inhibition of the activity of HMG-CoA reductase in the biosynthetic pathway of their cell membranes. In addition, this feed was characterized by an improved digestibility of dry matter by 13%. On the other hand, in the case of juveniles, the use of fermented feed supplements may have a positive effect on the growth and development of the animal. For example, the metabolites of the yeast *Saccharomyces cerevisiae* improve rearing parameters and accelerate the development of the rumen epithelium [37,38].

In a study by Shrivastava et al. [39] on wheat straw that was fermented with *Ganoderma sp. Rckk02* had a positive effect not only on the composition of the feed, but also on its consumption by animals was found. The *Ganoderma* sp. *Rckk02* showed improved digestibility, a significant decrease in the content of acid detergent fibers (ADF) and neutral detergent fibers (NDF), hemicellulose, lignin and cellulose after 15 days. During this period, there was also an increase in the metabolic energy and the amount of volatile fatty acids in the in the examined feed. The use of this fermented feed in goat nutrition increased the consumption of dry matter (DMI), digestible crude protein (DCP), total nutrient (TDN) and nitrogen (N) compared to the control group [39].

## 3. Probiotics and Prebiotics

The breeding of young ruminants is associated high expenditure (about 20% of the total costs). The use of antibiotics in order to prevent calves’ deaths, or to improve their growth in many countries, is prohibited or very limited [40]. An example of this is in the European Union, where the use of antibiotics and ionophores in animal production as growth promoters has been banned since 2006. It is related to the emerging antibiotic resistance of pathogenic bacteria, which may pose a threat to the health of both animals and humans. Therefore, there was a need to search for alternative methods that can be used therapeutically and prophylactically. Such alternatives are probiotics, prebiotics or a combination of them; synbiotics. Probiotics are defined as live microorganisms which, when consumed by humans or animals, have a beneficial effect on health through quantitative and qualitative effects on the intestinal microflora and/or modification of the immune system. They have recently become more and more popular as additives in animal nutrition, including both beef and dairy cattle, as alternatives to antibiotics [40,41,42].

The mechanism of probiotic effect on animal health is primarily based on the competition between bacteria recognized as beneficial and pathogens, and the replacement of pathogens by probiotic bacteria. On the other hand, the action of antibiotics is based on the elimination of all bacteria depending on the spectrum of its activity, both beneficial for the host and pathogenic microorganisms and may also lead to bacterial resistance [41,43].

Before the registration of a strain as a probiotic, its properties should be documented during clinical trials as follows [44]:

- Preservation of the ability to survive in the digestive system,
- Non-pathogenic and non-toxic,
- Taxonomic affiliation established with modern genetic methods (genus, species and strain),
- Show clinically documented beneficial effects on health,
- Be safe, i.e., show no undesirable side effects,
- Demonstrate stability and the possibility of large-scale production of biomass.

**Figure 2:**
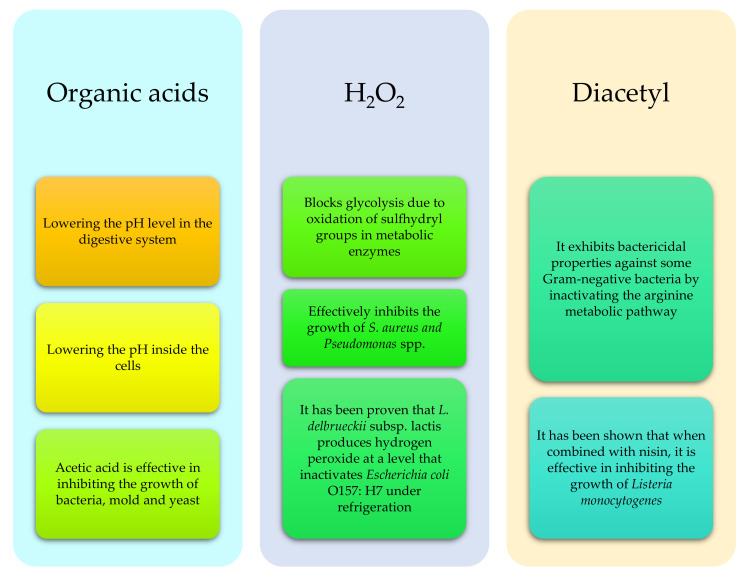
Selected antibacterial and bacteriostatic substances produced by probiotic microorganisms and their action in the digestive system of ruminants (based on: [40,41,42,57]).

The most commonly used types of bacteria in ruminant probiotics are: *Lactobacillus, Streptococcus, Entrococcus, Bacillus, Clostrididium*, some species of *Bifidobacterium, Propionibacterium, Megasphaera elsdenii* or *Prevotella bryantii* (in particular, Strain B14), as well as fungi such as *Saccharomyces—S. lipolytica* or *Asperillus* (*A. oryzae, A. niger*) [40,41,45]. In general, probiotic microorganisms are classified as lactic acid producing (LAB) bacteria, lactic acid (LUB) utilizing bacteria, yeast or other microorganisms (Table 1) [41]. The main function of probiotics is, among others, the production of a wide range of antibacterial and bacteriostatic substances such as: organic acids, bacteriocins, diacetyl, antibiotics and H2O2 [42,46] (Figure 2). They also compete for adhesion sites or nutrients in the digestive system. The ability to adhere to the epithelium, including the formation of a biofilm, allows their remaining on the intestinal wall, making it more resistant to peristalsis, thereby occupying a niche at the expense of potentially harmful microorganisms. In addition, they are involved in the production of amino acids or vitamins (e.g., B or PP vitamins), and also increase the pool of digestive enzymes (e.g., for α-galactosidases), which improves the digestion and assimilation of nutrients. Their use is also aimed at reducing the level of toxic products of metabolism in the gastrointestinal tract or detoxifying it, which helps to reduce the occurrence of diarrhea, e.g., in calves. They also stimulate the immune system by activating gut-associated lymphoid tissue (GALT), and modulate the activity of the immune system by reducing the activity of Th2 proallergic lymphocytes (allergy prophylaxis). In addition, some strains such as Propionibacterium thoenii T159 reduced methane production by up to 20%, and also increased VFA production by 21% [42,47,48,49,50,51]. The recent analyzes of probiotics on calves indicate that multi-component probiotics yield better results than single-component probiotics, due to their i interaction, with dosage also playing a vital role; it should be around 10^6^–10^7^ CFU/g per day [40,51]. Nowadays probiotics are increasingly combined with prebiotics and synbiotics. Prebiotics, being a component of a synbiotic, should have a beneficial effect on the digestive system of the animal and cooperate with probiotic bacteria. The use of prebiotics in cows, however, in particular in adult animals, is limited due to the possibility of degradation of most prebiotics in the rumen. Recent technological improvements allow the use of these compounds in beef and dairy cattle. Prebiotics are substances whose selective fermentation leads to specific changes in the composition or activity of the gastrointestinal microflora, in a beneficial way [52,53,54]. Prebiotic compounds include certain proteins, peptides, fats, and oligo—and polysaccharides such as cellooligosaccharides, inulin and lactulose. The aim of these substances is to act as a substrate for beneficial to health, naturally occurring bacterial strains in the gastrointestinal tract (Table 2) [55,56]. A synbiotic, i.e., the aforementioned combination of a probiotic and a prebiotic, is defined as a bioactive substance, or physiologically active food ingredient with a synergistic effect. This combination is intended to provide nutrients and a carrier for probiotic bacteria. Additionally, the action of such a combination should have a more intense effect on the animal’s organism [52,53].

Recent studies have shown a significant effect of prebiotics on feed efficiency and health status of ruminants—especially calves. The most commonly used prebiotics administered to calves are oligosaccharides (OS), which constitute the main group of prebiotics, and β-glucans [58].

## 4. Algae

Recently, an increase in the potential uses of algae as feed additives for ruminants has been observed. Ruminants capable of digesting unprocessed algae seem to be probably the best recipients of such supplementation, since in most cases their addition does not generate additional costs to improve their digestibility. In the case of ruminants, both micro, macroalgae and their extracts are used as feed additives [59,60,61,62]. As shown by the research conducted by Bulgariu et al. [63], the *Cladophora sericea* algae may be a potential carrier of metal ions necessary for the proper functioning of the ruminant system, including Cu (II), Co (II), and Zn (II). Unfortunately, during the implementation of the conducted research, it was reported that the desorption process of these ions was characterized by too low efficiency (>51%). In order to overcome this problem, the authors suggested the addition of activated carbon to the biomass in a 1:1 ratio. In this way, the efficiency of desorption is significantly increased, which, according to the authors, allows the effective use of algae as a feed additive. The above-mentioned method of supplementation, in which we administer both activated carbon and metal ions to ruminants together with algae, may, as shown by other studies, affect the composition of the microbiome of the digestive system of animals [64,65].

Another potential use of algae is related to the antibacterial activity of many species [66,67,68] and their extracts [69,70]. Their addition to feed can replace or reduce the amount of pharmacological agents used on animal farms. An additional argument in favor of such use of feed additives derived from algae is the recent increase in antibiotic resistance of many bacterial strains associated with large-scale animal husbandry [71,72,73].

Adequate algae supplementation may also be a response to the ever-increasing production of methane by the global ruminant population. As shown by recent studies, the addition of algae or their extracts to feed may reduce the production of methane by ruminants [60,61,62,74,75,76]. Abecia et al. [77] attributed the activity of these feed additives to the alteration of the rumen archaea population in favor of species that perform highly efficient processing of glucose into volatile fatty acids. Black et al. [76] agrees with this explanation of the process.

Choi et al. [62] showed that supplementation with extracts from five species of brown algae resulted in a statistically significant reduction in the amount of methanogenic archaea in the artificial rumen, which may be another explanation for the reduction in the amount of methane produced by way of emissions. However, as Choi et al. [60] have already pointed out, the observed effect of supplementation may be strongly related to the species that is the source of the administered extract, because in studies conducted on *Sargassum fusiforme* algae, the obtained effect was an increase in methane production, which may be explained by the presence of arsenic in the mentioned algae and its negative impact on the rumen microbiome.

However, in the case of supplementation with *Gelidium amansii algae*, Lee et al. [78] showed a statistically significant increase in the amount of methane produced, due to a raise of ciliate-dependent methanogens, while the number of methanogenic archaea was decreased. This proves This proves a strong need for further research aimed at full explanation and selection of species/extracts with the best predispositions to be used as feed additives reducing methane production by ruminants.

On the other hand, the reports in the available literature on the production of carbon dioxide during in vitro studies often present contradictory results confirming [79] or not [74] the observed decreasing trend. These discoveries and the appropriate control of these processes may be of great importance during the current period which the livestock industry is going through, therefore, it seems particularly important for the scientific community to focus on these issues.

The use of feed additives derived from algae may also have a negative effect on the composition of the milk of cows supplemented with them. In these cows, an increase in the amount of fat synthesis inhibitors in milk was observed. The phenomenon is possibly the result of the toxicity of very long-chain n-3 polyunsaturated fatty acids, for which the natural digestion of cows is not adapted [80].

## 5. Selected Additives of Plant Origin

Concerns about the use of antibiotics and other feed additives that may increase the resistance of pathogenic bacteria or burden the environment force reinforce the research for alternatives in animal husbandry. In addition to the previously mentioned possibilities, another option may be natural additives of plant origin, such as mixtures of essential oils or herbs (in the right amount—at high concentrations, they can act as toxic agents). They are used more and more often due to their properties that improve digestibility and feed quality. They are also added to modify the microbiota of the digestive system of ruminants, especially in terms of reducing the level of methane production. However, the operation of many of them requires further research [81]. 

Studies conducted so far on herbs such as oregano or rosemary have shown the effect of reducing the activity of protozoa, as well as reducing the production of methane [82,83]. Herbs are characterized by antioxidant capacity, metal ion chelation and quenching of singlet oxygen [84]. In a study, Kholif et al. [82] showed that the addition of rosemary (*Rosmarinus officinalis* L.) and lemongrass (*Cymbopogon citratus (DC.) Stapf.)* to the diet of Damascus goats during lactation increased the digestion of organic matter and also increased the concentration of VFA, propionate and glucose. On the other hand, the concentration of cholesterol in serum decreased, and milk yield of animals and the amount of fat and lactose in milk increased. Rosemary is a source of polyphenols (plant secondary metabolites), and it is characterized by antioxidant, anticancer and antibacterial properties [82,85]. Lemongrass, on the other hand, contains citral (eteric oil—non-annular terpenes), which is necessary for the synthesis of Vitamin A. It also has an antibacterial and antioxidant effect and reduces the activity of bacteria producing ammonia in the rumen [82,86]. In a study performed by Kolling et al. [83], where oregano and green tea extract were used in lactating cows, apparent digestibility coefficient for the mixture of both herbs was reduced. Both green tea extract and oregano reduced the production of methane by animals, and the addition of oregano also reduced the amount of fat in milk. Green tea (*Camellia sinensis* L.) is a source of polyphenols, as is the previously discussed rosemary, mainly catechins. It also contains saponins, caffeine and l-theanine. Thus, it exhibits antioxidant, antimicrobial, anti-coccidiosis and antiprotozoal effects [82,87,88].

Oregano (*Origanum vulgare*) is a source of essential oils like carvacrol and thymol (compounds from the group of terpenoids and phenols.). Both polyphenols contained in rosemary or green tea and components found in herbs in general can significantly affect the microbiome of the digestive system of ruminants, including the reduction of methanogenic archaea [81,89]. 

Essential oils, due to their natural origin and safety, are increasingly used to manipulate the microbiome, especially in the reduction of methane production in ruminants. Essential oils are volatile components of terpenoid and phenylpropanoid origin. Their composition may vary depending on the varieties of the same species or plant organs (leaves, stems, roots, flowers and fruits) and depending on the growing conditions, age or physiological state of the plant, as well as the extraction and processing method [81,89,90,91].

Belanche et al. [90] showed that the use of an essential oil mix (Agolin^®^ Ruminant) after a period of 4 weeks increased milk yield in dairy cows (+3.6%) compared to the control group. There was also a change in milk fat and protein content (+4.1%) and feed efficiency (+4.4%). The consumption of dry matter (−12.9%) and methane production (−8.8%) also decreased. Studies by Busquet et al. [92,93] also showed the effect of garlic essential oil (the composition mainly includes allyl-methyl and diallyl sulfide, allylmethyl and alkyl disulfide, dimethyl, allylmethyl and diallyl trisulfide) in reducing methane production. In contrast, studies by Garcia-Gonzales et al. [94] using thyme essential oil showed a reduction in cellulolytic bacteria.

The effect of essential oils is related to the biologically active substances contained in them. Most of these substances are bacteriostatic or bactericidal in order to protect plants against pathogens. These oils are lipophilic, and therefore, affect the cell membranes of bacteria, especially against gram-positive bacteria. However, some substances in essential oils can also affect gram negative bacteria due to their small size that can penetrate the membrane and damage it. Some substances contained in essential oils can also cause the coagulation of cytoplasmic material, thanks to which they can affect not only the level of bacteria, but also weaken the growth of fungi, protozoa and viruses. In the case of the impact on methanogenic archaea, it is related to their action, which impairs energy metabolism [81,89].

Another alternative for plant products is the use of residues or by-products such as e.g., nut shell fluids. One such additive is cashew nut shell fluid (CNSL). It is a by-product of cashew production, often used in the production of paints, varnishes, coatings, brake linings and others [95]. A study by Konda et al. [96] on Thai cattle and marsh buffalos using CNSL as a feed additive showed a reduction in methane production potential of 53% and 73% in Thai cattle and marsh buffalos, respectively, after four weeks of feeding. There was also a decrease in the level of protozoa, an increase in the number of bacteria of the genus *Prevotella*, as well as changes in the structure of methanogenic archaea. However, no effect on feed intake or rumen pH was found. Similar results in the studies were also obtained by Maeda et al. [20]. The experiment was carried out on Lai Sind beef cattle. This study demonstrated the effect of the CNSL addition to reduce methane emissions by about 20%, with no apparent adverse effects on feed intake and digestibility. Similar increase in propionate amounts has been reported in study by Konda et al. [96], where reduction in the relative abundance *of Methanobacteriales* has been observed, indicating a direct inhibitory effect of CNSL on methanogens.

## 6. Summary 

Ruminant nutrition and the use of feed additives can be important and meaningful ways in the manipulation of the microbiome. It also allows reducing the negative impact of animals on the environment—mainly through the production of methane. Assessing the effects of various types of natural feed additives has recently become the main alternative to antibiotics, the use of which is increasingly limited. On the other hand, the use of additives of natural or probiotic origin is assessed as safe for humans and animals.

## Figures and Tables

**Table 1:** Classification of probiotic microorganisms (according to Retta [41]).

Classification	Example
LAB	*Lactobacilli*
	*Enterococci*
LUB	*Megasphaera elsdenii*
Yeats	*S. cerevisiae*
	*Y. lipolytica*
Other	*A. oryzae*
	*A. niger*

LAB—Lactic Acid Bacteria; LUB—Lactic Acid Utilizing Bacteria.

**Table 2:** Selected prebiotic substances and their action in the digestive system of ruminants [own study based on [56,58].

Prebiotic	Effect on Absorption Nutrients	Impact on Health
Fructooligosaccharides (FOS)	Improving feed efficiency, increasing yields	Positive effect on the immune system of calves and reduction of mortality rates.
Galactooligosaccharides (GOS)	Not fully known—divergent research results	Probably beneficial effects on LAB in the digestive system—however, further research is required
Mannanoligosaccharides (MOS)	There was an increase in the length of the rumen papillae and the height of the jejunum villi, which was probably related to the increase in substrate availability by MOS-utilizing bacteria.	Positive effect on health in calves—reduction of the intensity of diarrhea
Cellooligosaccharides (CO)	Acceleration of intestinal development, improves feed efficiency, increased calf growth.	Health and microbiological measurements do not change

## Data Availability

Not applicable.
